# Splicing factor-modulated generation of mechano growth factor regulates physiological processes in osteoblasts under mechanical stimuli

**DOI:** 10.1080/19336918.2019.1686103

**Published:** 2019-11-02

**Authors:** Qian Yi, Huan Liu, Jianguo Feng, Yanjiao Wu, Weichao Sun, Mengting Ou, Liling Tang

**Affiliations:** a Key Laboratory of Biorheological Science and Technology, Ministry of Education, College of Bioengineering, Chongqing University, Chongqing, China; b Department of Physiology, School of Basic Medical Sciences, Southwest Medical University, Luzhou, Sichuan, China; c Department of Orthopaedics, Affiliated Traditional Chinese Medicine Hospital, Southwest Medical University, Luzhou, Sichuan, China; d Department of Anesthesiology, The Affiliated Hospital of Southwest Medical University, Luzhou, Sichuan, China

**Keywords:** Mechanical stimuli, MGF, osteoblasts, Erk1/2 signal pathway, ASF/SF2 proteins

## Abstract

Mechanical stimuli influence various physiological processes in osteoblasts. We previously showed that mechano-growth factor (MGF), a splicing variant of insulin-like growth factor 1, is highly expressed in osteoblasts in response to mechanical stimuli. This study aims to explore the systemic functions of MGF in osteoblasts, and the mechanisms by which mechanical stress regulates the alternative splicing of *Igf1* to generate MGF. We found that MGF promoted the proliferation and migration of osteoblasts, while it inhibited their differentiation via Erk1/2 pathway. Furthermore, cyclic stretching upregulated the expression of ASF/SF2, which in turn regulated the expression of MGF. Our findings indicate that mechanical stimuli influence the physiological responses of osteoblasts by increasing the expression of MGF, which is regulated by splicing factors.

## Introduction

Mechanical cues play important roles in cell proliferation and differentiation, especially in bone development [1,2]. Osteoblasts can sense mechanical signals and transform them into biological responses, such as an increase of cell number, reorganization of the cytoskeleton, and secretion of extracellular matrix [3–6].

**Figure 1.**
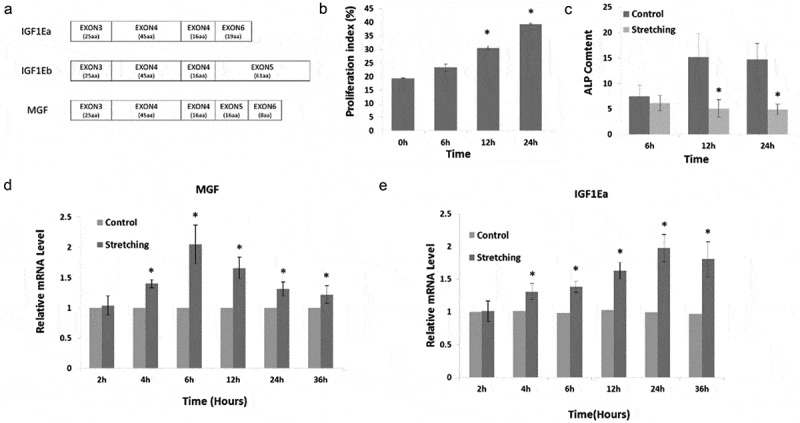
Physiological effects and MGF expression in response to cyclic stretching with a magnitude of 15% and frequency of 30 cycles/min in osteoblasts. (a). The diagram of alternative splicing variants of IGF1. (b) Proliferation of osteoblasts in response to cyclic stretching with a magnitude of 15% and frequency of 30 cycles/min for 6, 12, and 24 h. (c) ALP content of osteoblasts in response to mechanical stretching. (d) MGF expression. (e) IGF1Ea expression. The data are the mean ± SD, n = 3, *p < 0.05.

Alternative splicing is an important post-transcriptional mechanism that contributes to proteomic diversity [7–9]. Many kinds of extracellular stimuli regulate alternative splicing, including heat, stress, ultraviolet radiation, genotoxic, and chemical stimuli. However, relatively few studies have examined the regulation of alternative splicing induced by mechanical stress. To date, some mechanical stress-induced genes have been reported to have multiple splicing variants, including vascular endothelial growth factor, tension-induced/inhibited proteins, versican, CD44, serum response factor, and insulin like growth factor 1 (IGF1) [10–16]. IGF1 has three splicing variants, namely, IGF1Ea, IGF1Eb, and IGF1Ec [14–17]. The differences between the alternative splicing variants of IGF1 (Figure 1(a)) mainly reside in alternative exons encoding C-terminal peptides (E-peptides). The three E-peptides of human IGF1 (hEa, hEb, and hEc) are encoded by exons 4/6, 4/5, and 4/5/6, respectively. IGF1Ec is also named mechano growth factor (MGF) as it was initially reported as a mechano-sensitive factor. One distinctive feature of MGF is that a 49 base pair insert is added between exons 4 and 6, which leads to a reading frame shift resulting in the unique 24-amino-acid C-terminus of hEc. Although, previous studies have shown that mechanical stimuli are involved in the regulation of the alternative splicing process of the *Igf1* gene, it is still unclear about how mechanical stress regulates alternative splicing to generate MGF, and the function of the full-length splicing variant of MGF in osteoblasts is also unknown.

In this study, we investigated the physiological effects of mechanical stress on osteoblasts and examined the expression of MGF in response to different periods of stretching in osteoblasts. Our results indicated that mechanical stress significantly promoted proliferation, while it inhibited alkaline phosphatase (ALP) activity in osteoblasts. In addition, MGF expression was also upregulated in response to mechanical stress, indicating that mechanical stimuli are involved in the regulation of alternative *Igf1* gene splicing. Then, we investigated the physiological effects of MGF on osteoblasts and the roles of serine/arginine (SR) proteins in the regulation of MGF to characterize the mechanism of a splicing model under mechanical stimuli. Our results suggested that MGF promoted cell proliferation and migration, while it inhibited differentiation via Erk1/2 signal pathway. In addition, SR proteins were involved in alternative *Igf1* gene splicing events to generate the alternative splicing variant MGF under mechanical stimuli.

## Materials and methods

### Cell culture, transfection, and peptide synthesis

A tissue pieces-stick method was used to harvest osteoblasts from newborn Wistar rat calvaria. Calvaria were dissected and placed in 50-cm^2^ flasks and cultured in Dulbecco’s modified Eagles medium/F12 (HyClone, Logan, UT) supplemented with 10% foetal bovine serum (HyClone, Logan, UT) and antibiotics (100 IU/mL penicillin, 100 μg/mL streptomycin). These flasks were incubated bottom up at 37°C in a humidified atmosphere of 5% CO_2_. After culturing for 4–6 h, the culture flasks were inverted. When cell confluence reached 80%, the osteoblasts were digested with 0.25% trypsin containing EDTA and purified. Von Kossa and ALP staining were used to identify these purified osteoblasts, and positive cells from the second to fourth passages were used in all experiments.

The osteoblasts were transfected with a lentiviral plasmid carrying green fluorescent protein (GFP) and SR-rich splicing factor 1 (ASF/SF2) or SR-rich splicing factor 2 (SC35), or a lentiviral plasmid carrying GFP only. The transfected cells were named ASF/SF2-GFP-osteoblasts, SC35-GFP-osteoblasts, or GFP-osteoblasts, respectively. At 72 h later, western blot analysis was utilized to select stably transformed transgenic cells.

MGF peptide synthesis was performed as described previously [18]. To prepare GST fusion proteins, pGEX-4T-1 vectors containing the coding sequence for MGF were transformed into BL21 (DE3) competent cells. GST or GST-fusion proteins were purified using a glutathione-Sepharose 4B column following the manufacturer’s instructions (GE Healthcare, Pittsburgh, USA).

### Cell cyclic stretching device and mechanical stimulation

A cyclic stretching device was used to apply cyclic stretching to osteoblasts [19]. The device consisted of sliding rails, sliding support, motor, base, and culture box. The cells were seeded at a density of 2.0 × 10^4^ cells/cm^2^ on a silicone membrane surface fixed in the culture box of this device. Stretched stimulation was administered to the seeded cells through the deformation of the silicone membrane. In this study, the magnitude of strain was 15% with a frequency of 30 cycles/min. The culture conditions of the stretched cells were the same as for the control cells.

### Cell cycle assay

After stretching, the cells were washed with phosphate-buffered saline and digested. Test cells were immobilized in 75% alcohol and stained with propidium iodide (Sigma-Aldrich, St. Louis, MO). A flow cytometer (FACSCalibur; Becton Dickinson, Franklin Lakes, NJ) was used for single-cell analysis.

### Cell counting kit-8 assay

CCK-8 assay was used to detect cell proliferation and cytotoxicity. Briefly, after treatment, the medium was removed and 100 μL fresh medium with 10 μL Cell Counting Kit-8 (CCK-8; Solarbio, Beijing, China) was added to each well of a 96-well plate. The plate was incubated for 2 h in a CO_2_ incubator and absorbance was measured at 450 nm using a microplate reader (Bio-Rad Laboratories, Hercules, CA).

### ALP assay

An ALP Assay Kit (Nanjing Jiancheng Bioengineering Institute, Nanjing, China) was used to perform quantitative ALP measurements. Briefly, after treatment, the cells were harvested and washed. The cells were resuspended in 50 µL assay buffer. ALP enzyme activity was analysed according to the manufacturer’s instructions. Enzyme activity was quantified by absorbance measurements at a wavelength of 405 nm on a microplate reader (Bio-Rad Laboratories) and calculated according to a series of p-nitrophenol standards (U/mL).

### Wound healing assay

Osteoblasts were seeded into a 12-well plate for 24h, and then scratched and treated with 0–100 ng/mL MGF peptide. Photomicrographs were taken at 0h, 3h, 6h and 24h, and the wound healing ratio was measured using Image J.

### Transwell assay

The migration of osteoblasts was tested using a 24-well Transwell plate inserted with 8 mm pores (Coring Co-Star, Shanghai, China). When cells were 90% confluent, they were subjected to serum deprivation for 12 h. Then, cells (5*10^4^) were added to the upper chamber. For studying the inhibitor effect of Erk1/2, the cells were treated with PD98059 (50 mM) and U0126 (10 mM) for 30 min respectively before adding into the upper chamber. The concentrations of MGF peptide in bottom well were 0, 10, 50 and 100 ng/mL respectively. The cells migrated for 5 h and then the non-migrated cells remaining in the upper chamber were removed with a cotton bud. The migrated cells were stained with 0.1% crystal violet for 30 min.Pictures of the cell layers were obtained using were analysed light microscope and the cell numbers using Image J software.

### Transfections and RNA interference assay

All transfections were performed using LipoRNAiMAX (Life Technologies), according to the manufacturer’s instructions. Small interfering RNA (siRNA) duplexes were transfected at 20 nM. The sequences for each siRNA are listed in Table 1. The efficiency of RNA interference was evaluated by RT-PCR.

**Table 1.** Small interfering RNA sequences.

Gene	Sequence
ASF/SF2	GAAAGAAGATATGACCTATTT
SC35	GCGUCUUCGAGAAGUACGGTT

### RNA isolation

Total RNA was isolated from control and treated cells using a High Pure Viral RNA Kit (Bioteke Corporation, Beijing, China). RNA integrity was determined by electrophoresis on a 1.5% agarose gel. RNA quality and quantity were determined using a spectrophotometer (NanoDrop 2000; Thermo Fisher Scientific, Waltham, MA).

### Semi-quantitative RT-PCR

Total RNA was used to synthesize cDNA with a PrimeScript^TM^ RT Reagent Kit (Perfect Real Time; TaKaRa). A total volume of 10 μL, containing 2 μL of 5 × PrimeScript^TM^ Buffer, 0.5 μL PrimeScript^TM^ RT Enzyme Mix I, 0.5 μL Oligo dT Primer (50 μM), 0.5 μL Random 6-mers (100 μM), 2–3 μL RNA, and 3.5–4.5 μL RNase-free dH_2_O, was incubated at 37°C for 15 min, followed by inactivation at 85°C for 5 s.

The expression of osteoblast-specific genes was determined by semi-quantitative RT-PCR in a thermo cycler (Bio-Rad). A total volume of 5 μL cDNA template, 0.25 μL TaKaRa Taq, 5 μL of 10× PCR Buffer, 3 μL MgCl_2_, and 4 μL dNTP Mixture was added to a final volume of 50 μL. The following thermal cycling conditions were used: 30 cycles at 94°C for 30 s, 55°C for 30 s, and 72°C for 1 min, and a final step at 72°C for 5 min. The forward and reverse primers for each transcript are listed in Table 2. The sequences of the differentially expressed genes were obtained from GenBank. Primers were designed by Primer 3.0.

**Table 2.** Sequences of the primers used for semi-quantitative RT-PCR.

Gene	Forward (5′–3′)	Reverse (5′–3′)
ALP	TGACTGACCCTTCCCTCTCG	TCAATCCTGCCTCCTTCCAC
RUNX2	CTTCAAGGTTGTAGCCCTCGGA	GAGTAGTTCTCATCATTCCC
COL1	CCAGCTGACCTTCCTGCGCC	CGGTGTGACTCGTGCAGCCA
OPN	CCTCTGAAGAAACGGATGACT	CTGTGTGTTTCCACGCTT
MGF	CGAAGTCTCAGAGAAGGAAAGG	ACAGGTAACTCGTGCAGAGC
ASF/SF2	GGAAGACGCGGTGTATGGTC	CACCTGCTTCACGCATGTG
SC35	CCCGATGTGGAGGGTATGAC	GAGACTTCGAGCGGCTGTAG
β-actin	ATATCGCTGCGCTGGTCGTC	AGGATGGCGTGAGGGAGAGC

### Quantitative real-time PCR

The differentially expressed genes were validated by quantitative real-time PCR on a thermo cycler (Bio-Rad Laboratories). A total volume of 2 μL cDNA template and 12.5 μL of 2 × SYBR Premix ExTaq^TM^ II was added to a final volume of 25 μL. The following thermal cycling conditions were used: 95°C for 30 s, and 40 cycles at 95°C for 5 s and 60°C for 30 s. The forward and reverse primers for each transcript are listed in Table 3. The sequences of the differentially expressed genes were obtained from GenBank. Primers were designed by Primer 3.0.

**Table 3.** Sequences of the primers used for qRT-PCR.

Gene	Forward (5′–3′)	Reverse (5′–3′)
MGF	GGAGGCTGGAGATGTACTGTGCT	TCCTTTGCAGCTTCCTTTTCTTG
ASF/SF2	GTTCGAGGACCCGCGAGACG	GGATGGCGGGCCATAGCGG
SC35	ATCCCGCTCGAGGTCCAGGT	GCGGCTTGCCGATCCATCATTC
ALP	CATTTGTGCCAGAGAAAGAGAA	GTTGGTGTTGAGTTTTTGGAGTT
RUNX2	CCATAACGGTCTTCACAAATCC	GCGGGACACCTACTCTCATACT
COL1	TTCCCGGTGAATTCGGTCTC	ACCTCGGATTCCAATAGGACCAG
OPN	CTTTCACTCCAATCGTCCCTAC	CTGCCCTTTCCGTTGTTGTC
β-actin	GGAGATTACTGCCCTGGCTCCTA	GACTCATCGTACTCCTGCTTGCTG

### Western blotting analysis

Osteoblasts were lysed in RIPA buffer (Beyotime, Shanghai, China) with a proteinase inhibitor or PMSF at a final concentration of 1 mM. The samples were incubated on ice for 30 min, centrifuged at 14,000 × *g* for 3–5 min at 4°C, and the supernatant was collected. Protein concentration was detected by a BCA kit (Beyotime) according to the manufacturer’s instructions. Proteins were resolved by sodium dodecyl sulphate-polyacrylamide gel electrophoresis in 10% polyacrylamide gels and transferred to polyvinylidene difluoride membranes (Millipore, Burlington, MA). The membranes were blocked in Tris-buffered saline with 0.1% Tween and 5% skim milk for 1 h at room temperature. The membranes were immunoblotted with primary antibodies (1:1000; Santa Cruz Biotechnology, Santa Cruz, CA) overnight at 4°C, and specific IgG antibodies (ZSGB-BIO, Beijing, China) for 2 h at room temperature. Immunoreactive proteins were visualized via chemiluminescent detection (BeyoECL Plus; Beyotime), and relative band intensity was determined by using Quantity One software.

### Statistical analysis

All data are presented as the mean ± standard deviation (SD) from at least three independent experiments. Comparisons between two groups were performed by the two-tailed Student’s t test; p < 0.05 was considered significant.

## Results

### Physiological effects of mechanical stimuli on osteoblasts

In order to investigate the proliferation capacity of osteoblasts under stretching, flow cytometry and propidium iodide staining were used to detect DNA content and cell cycle phases in osteoblasts in response to cyclic stretching with a magnitude of 15% and frequency of 30 cycles/min for 6, 12, and 24 h. Mechanical stress changed the cell cycle distribution of osteoblasts significantly after stretching for 12 and 24 h, resulting in the accumulation of cells in the G2/M and S phases (Table 4). Cell proliferation was also examined by the proliferation index (Figure 1(b)). In response to cyclic stretching, the proliferation index increased at 12 and 24 h (p < 0.05). These results suggested that the pro-proliferation activity of mechanical stress was induced by changing the pattern of cell cycle progression.

**Table 4.** Cell cycle and proliferation index of osteoblasts in response to mechanical stretching.

	Cell cycle (%)			
Group	G1/G0	G2//M	S	Proliferation index
Control	80.64 ± 0.09	8.39 ± 2.35	10.95 ± 2.25	19.34 ± 0.11
6 h	76.69 ± 1.39	9.93 ± 1.23	13.39 ± 0.16	23.32 ± 1.39
12 h	69.40 ± 0.57*	14.30 ± 2.82*	16.30 ± 2.26*	30.60 ± 0.57*
24 h	60.65 ± 0.36*	14.97 ± 0.75*	24.44 ± 1.04*	39.41 ± 0.29*

The ALP activity of osteoblasts under cyclic stretching was detected by an ALP assay. Mechanical stress suppressed ALP activity in osteoblasts. This effect was more significant after stretching for 12 and 24 h compared to cyclic stretching for 6 h (Figure 1(c)).

### MGF expression in osteoblasts in response to stretching for different periods of time

In the present study, we found that MGF expression was increased in osteoblasts in response to cyclic stretching with a magnitude of 15% and frequency of 30 cycles/min for 2, 4, 6, 12, 24, and 36 h (Figure 1(d)). qRT-PCR showed that MGF expression was increased significantly after stretching for 4 h. MGF expression was higher after 6 h stretching than after 4 h stretching, and the expression of MGF was then decreased when the cells were stretched for longer than 6 h (Figure 1(d)). That is, the increase of MGF expression by cyclic stretching was more pronounced in response to 6 h stretching. However, IGF1Ea expression was more pronounced in response to 24 h stretching (Figure 1(e)).

### MGF overexpression increases osteoblast proliferation, but inhibits osteogenic differentiation

**Figure 2.**
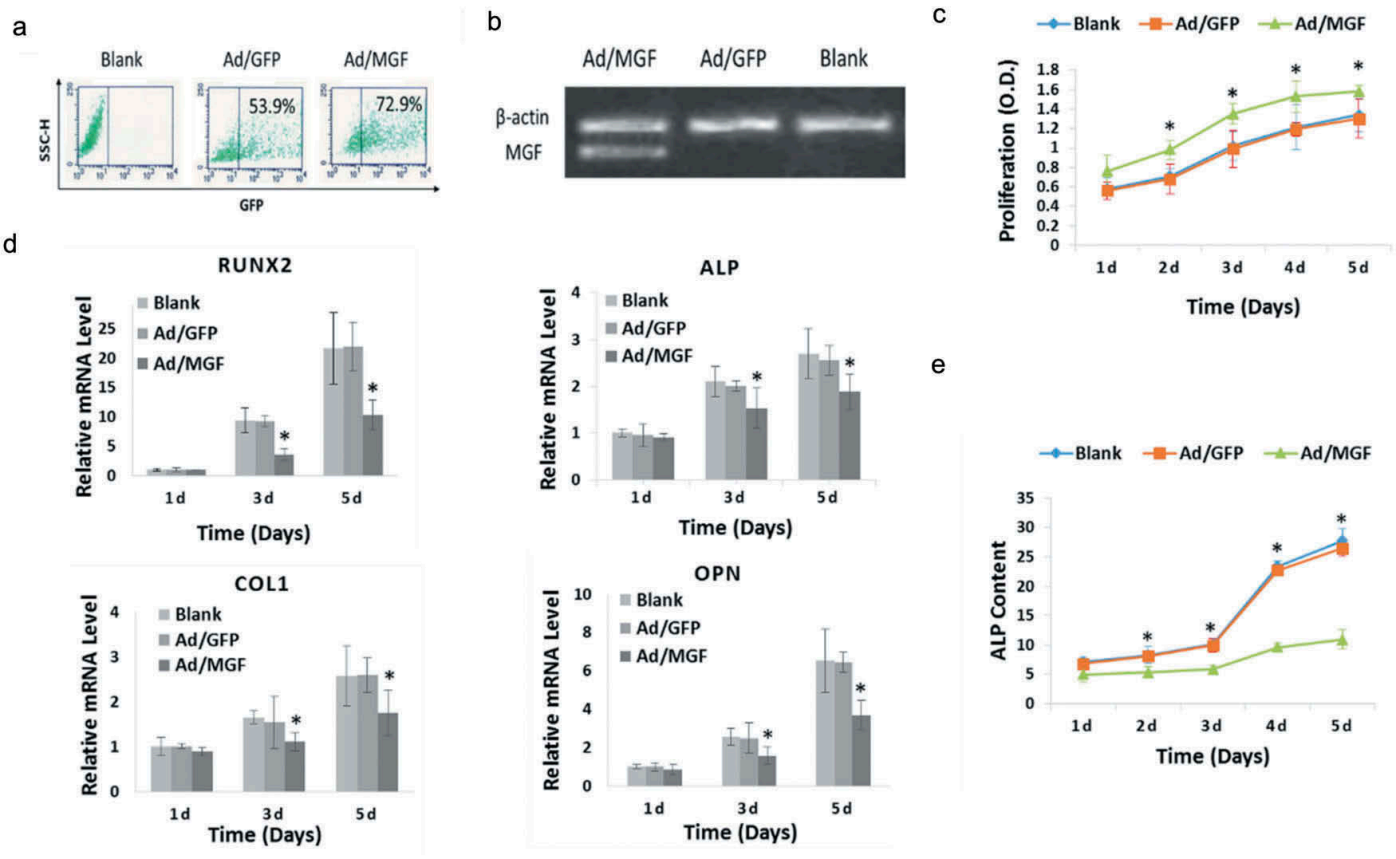
Physiological effects of MGF overexpression on osteoblasts. (a) GFP expression in osteoblasts assessed by flow cytometry. (b) MGF expression in osteoblasts assessed by RT-PCR. (c) Proliferation of osteoblasts in response to MGF overexpression. (d) Expression of osteogenic-specific genes in osteoblasts in response to MGF overexpression. (e) ALP content of osteoblasts in response to MGF overexpression. The data are the mean ± SD, n = 3, *p < 0.05.

To investigate the effects of MGF on osteoblast proliferation and differentiation, we constructed a recombinant adenovirus vector containing the *MGF* gene (Ad/MGF) and transfected this vector into osteoblasts. Flow cytometry results shown that transfection efficiency of Ad/MGF was 72.9% in osteoblasts (Figure 2(a)). Osteoblasts overexpressed *MGF* mRNA after transfection with Ad/MGF, while no exogenous *MGF* mRNA was detected in non-transfected cells (blank group) and Ad/GFP-transfected cells (Figure 2(b)).

Cell proliferation was analysed using a CCK-8 assay. Optical density was measured at 1–5 d after plating. No significant difference in cell proliferation was observed between Ad/GFP-transfected cells and the blank group, which indicated that transfection with Ad/GFP did not induce cytotoxicity. In addition, in Ad/MGF cells, there was a significant increase in cell proliferation at 1–5 d after plating compared to Ad/GFP-transfected cells (Figure 2(c)).

*ALP*, runt-related transcription factor 2 (*RUNX2*), collagen type 1 (*COL1*), and osteopontin (*OPN*) are osteogenic-specific genes expressed by osteoblasts [20]. To investigate the effects of Ad/MGF on osteogenic differentiation, the expression of osteogenic genes was measured. Quantitative real-time-PCR analysis showed that transfected with Ad/MGF downregulated the mRNA levels of these osteogenic-specific genes on day 3 and day 5 (Figure 2(d)). Then, ALP activity was measured. It’s shown that in the blank and Ad/GFP groups, ALP activity was increased gradually from 1–5 d. However, compared with the Ad/GFP group, when the osteoblasts were transfected with Ad/MGF, ALP activity was significantly decreased on days 1–5 to 21.7%, 32.0%, 41.7%, 61.6%, and 59.0%, respectively (Figure 2(e)).

### MGF peptide increases osteoblast proliferation and migration, but inhibits osteogenic differentiation

**Figure 3.**
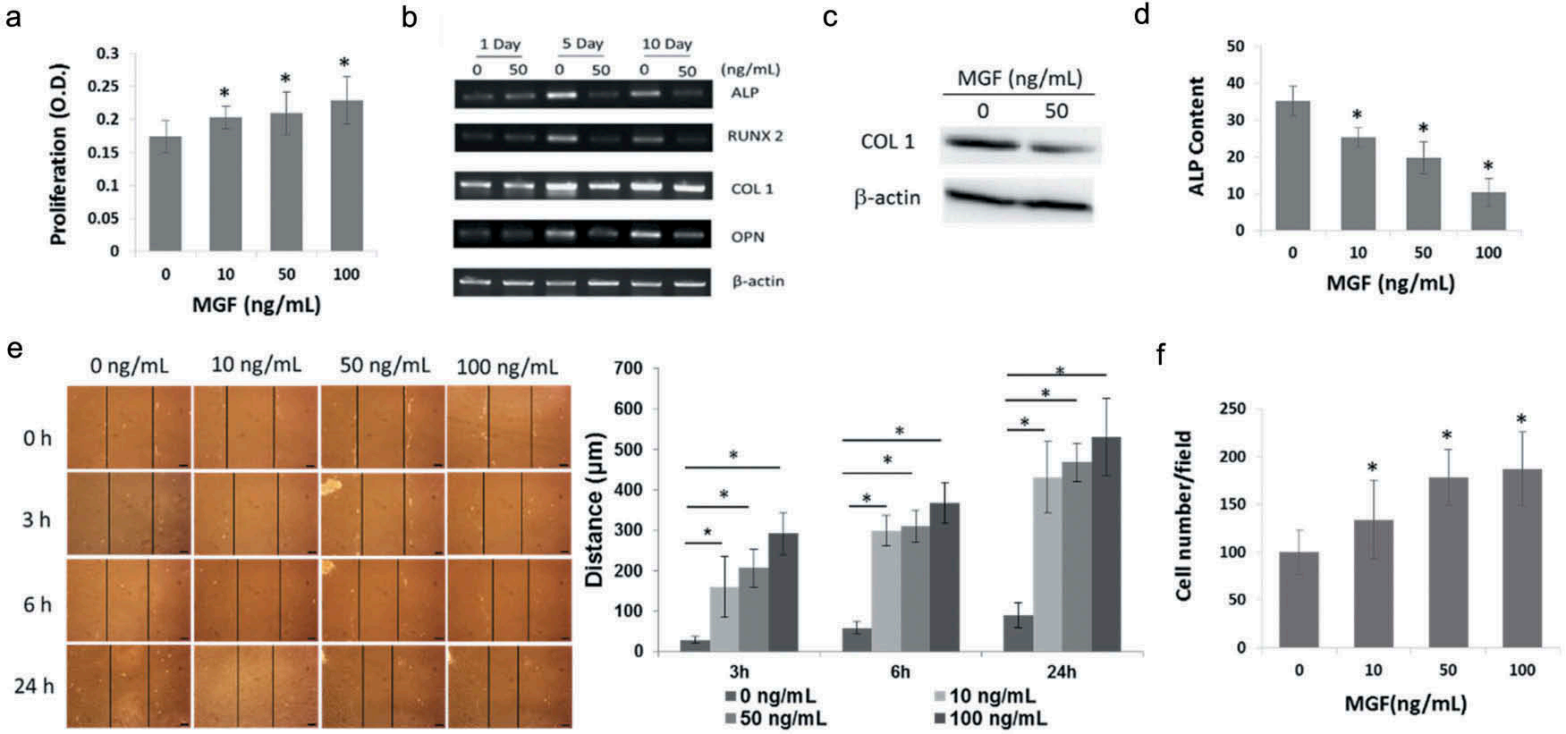
Physiological effects of different concentrations of MGF peptide on osteoblasts. (a) Proliferation of osteoblasts in response to different concentrations of MGF peptide at 48 h. (b) Expression of osteogenic-specific genes in osteoblasts in response to different concentrations of MGF peptide on day 5 and day 10. (c) Protein level of COL1 in osteoblasts in response to MGF peptide (50 ng/mL) on day 5. (d) ALP content of osteoblasts in response to different concentrations of MGF peptide on day 5. (e) Migration of osteoblasts in response to different concentrations of MGF peptide at 3h–24h by wound healing assay. (f) Migration of osteoblasts in response to different concentrations of MGF peptide at 5h by transwell assay. The data are the mean ± SD, n = 3, *p < 0.05. Bars, 100 μm.

In order to demonstrate that the increase of cell proliferation was due to the effects of MGF, the osteoblasts were treated with different concentrations of a synthetic MGF peptide. Cell proliferation was increased by treatment with the MGF peptide at 10–100 ng/mL for 48 h (Figure 3(a)).

In order to confirm that the inhibition of osteogenic differentiation was due to the effects of MGF, the expression of these genes and ALP activity were tested following the treatment of osteoblasts with the synthetic MGF peptide. RT-PCR analysis showed that treatment with 50 ng/mL MGF peptide downregulated the mRNA levels of these osteogenic-specific genes on day 5 and day 10 (Figure 3(b)). Western blot results showed that treatment with 50 ng/mL MGF peptide downregulated the protein levels of COL 1 on day 5 (Figure 3(c)). In line with the RT-PCR data, ALP activity was significantly decreased on day 5 following treatment with 10–100 ng/mL MGF peptide (Figure 3(d)). These results indicated that MGF peptide potently inhibited the differentiation of osteoblasts *in vitro*.

In order to demonstrate that the increase of cell migration was due to the effects of MGF, wound healing assay was performed following the treatment of osteoblasts with the synthetic MGF peptide. The migratory distance of osteoblasts was significantly increased after 3h-24h following treatment with 10–100 ng/mL MGF peptide (Figure 3(e)). In line with the wound healing assay data, transwell assay results showed that the numbers of cells in the lower surface of the menbrane were also significantly increased after 6h following treatment with 10–100 ng/mL MGF peptide (Figure 3(f)).

### MGF peptide regulates osteoblasts proliferation, migration and differentiation via Erk1/2 signal pathway

**Figure 4.**
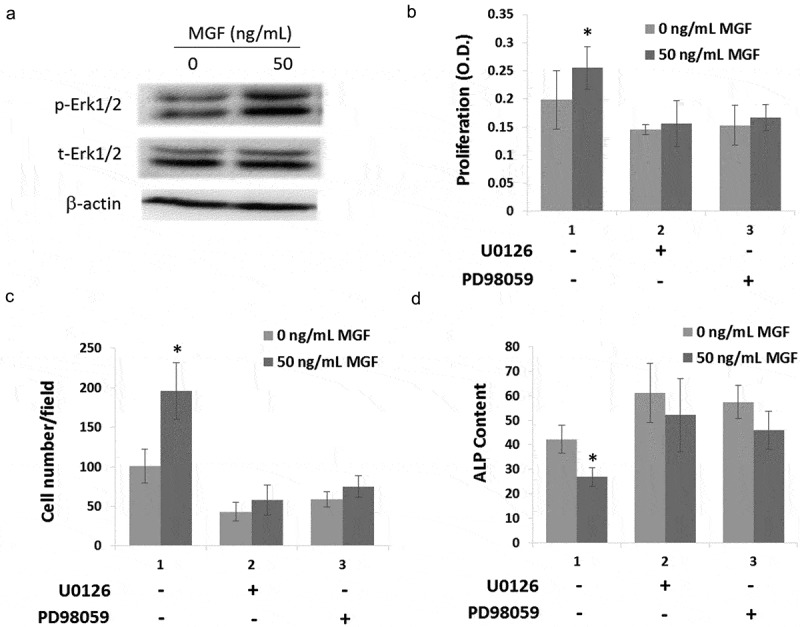
MGF peptide regulates osteoblasts proliferation, migration and differentiation via Erk1/2 signal pathway. (a) The expression level of phosphorylated Erk1/2 in osteoblasts in response to MGF peptide (50 ng/mL) for 30 mins. (b–d) The proliferation, migration and differentiation of osteoblasts in response to MGF peptide and Erk1/2 inhibitors. The data are the mean ± SD, n = 3, *p < 0.05.

In order to investigate the mechanism of MGF on osteoblast proliferation, migration and differentiation, the phosphorylation of Erk1/2 was measured. Western blot results showed that treatment with 50 ng/mL MGF peptide increased the expression of phosphorylated Erk1/2 (Figure 4(a)).

In order to demonstrate that the effects of MGF on cell proliferation, differentiation and migration were due to the activity of Erk1/2 signal pathway, the inhibitors of Erk1/2 (U0126 and PD98059) were used following the treatment of osteoblasts with the synthetic MGF peptide. Cell proliferation and migration were not increased by treatment with the MGF when cells were pretreated with U0126 and PD98059 (Figure 4(b,c)). And cell differentiation was also not inhibited by treatment with the MGF when cells were pretreated with U0126 and PD98059 (Figure 4(d)).

### ASF/SF2 regulates MGF expression under mechanical stimuli

**Figure 5.**
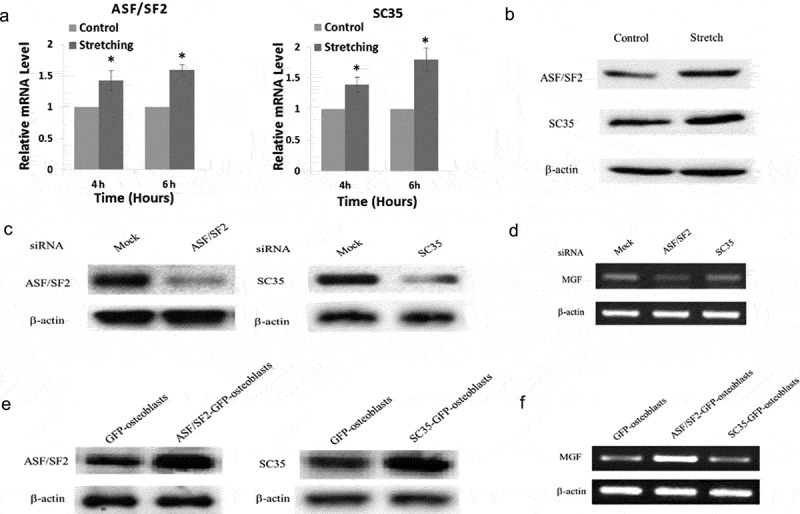
Regulation of MGF expression by ASF/SF2 under mechanical stimuli. (a) mRNA expression of the splicing factors ASF/SF2 and SC35 in osteoblasts in response to cyclic stretching for 4 and 6 h. (b) Protein level of ASF/SF2 and SC35 in osteoblasts in response to stretching for 4 h. (c) ASF/SF2 and SC35 expression in ASF/SF2-knockdown or SC35-knockdown osteoblasts. (d) mRNA expression of MGF in ASF/SF2-overexpressing or SC35-overexpressing osteoblasts. (e) Expression of ASF/SF2 and SC35 in ASF/SF2-knockdown or SC35-knockdown osteoblasts. (f) mRNA expression of MGF in ASF/SF2-overexpressing or SC35-overexpressing osteoblasts.

In the present study, we measured the expression of SR proteins at the mRNA and protein level in response to cyclic stretching. We found that ASF/SF2 and SC35 expression was upregulated significantly in osteoblasts in response to cyclic stretching with a magnitude of 15% and frequency of 30 cycles/min for 4 and 6 h (Figure 5(a,b)), which indicated that SR proteins were regulated by mechanical stress.

Since the expression of MGF and splicing factors was regulated by mechanical stress, we examined whether ASF/SF2 or SC35 affected MGF expression under mechanical stress. For this purpose, we first knocked down ASF/SF2 or SC35 expression by siRNA (Figure 5(c)) and analysed the mRNA expression level of MGF. Our results showed a downregulation of endogenous MGF mRNA expression in ASF/SF2-knockdown osteoblasts, but there was no obvious change in MGF mRNA expression in SC35-knockdown osteoblasts (Figure 5(d)). When we overexpressed ASF/SF2 or SC35 in osteoblasts (Figure 5(e)), the expression of MGF mRNA was upregulated in ASF/SF2-overexpressing osteoblasts, but it did not change significantly in SC35-overexpressing osteoblasts (Figure 5(f)).

## Discussion

Mechanical stimuli are important factors in the regulation of bone modelling and remodelling [21–24]. Previous studies found that bone cells can respond to various types of mechanical stimulation, including stretching, fluid flow, and hydrostatic pressure [25–31]. Osteoblasts are the major functional component of bone. To date, many investigators have studied the responses of osteoblasts to mechanical stimuli *in vitro* and found that mechanical stimuli may contribute to the overall anabolic response of osteoblasts [21,32–34]. The findings reported here demonstrated that cyclic stretching regulated the viability of osteoblasts by influencing the pattern of cell cycle progression and inhibiting osteoblast differentiation at an early stage.

When a cell senses mechanical stimuli, a large number of cellular events are activated, such as modification of chromatin structure, alteration of gene expression, and change of protein synthesis. Alternative splicing is an important post-transcriptional modification that is able to increase the diversity of mRNA transcripts and then contributes to the complexity of proteins. IGF1 responds to mechanical stimuli by generating different protein isoforms via alternative splicing. In addition, MGF, as a mechanical response factor, is upregulated rapidly under mechanical stimuli [14–17]. Our findings reported here also demonstrated that MGF was upregulated in osteoblasts under cyclic stretching, which indicated that mechanical stimuli are involved in the regulation of *Igf1* alternative splicing to generate MGF.

Comparing the structures of the IGF1 alternative splicing variants, the differences are located in their C-terminal E-peptides. In addition, the expression patterns and functions of MGF E peptides in different tissues have been studied by many investigators. However, to date, MGF E peptides have not been detected in a physiological state. Therefore, the functions of full-length MGF peptide also need to be investigated, which will help us to understand the systemic role of MGF. Previous studies found that MGF is highly expressed in many tissues, such as skeletal muscle, heart, osteoblast, tendon, and brain, in response to damage, stretching, and overloading; however, the function of MGF has not been clarified fully in these tissues. In the present study, the effects of MGF on the proliferation, migration, and differentiation of osteoblasts were examined to clarify further the function of MGF and to understand its role in regulating bone growth, development, and injury repair. Our results showed that MGF has a passive effect on osteoblast proliferation and migration, while it has an inhibitory effect on osteoblast differentiation via Erk signal pathway. As MGF is highly expressed in osteoblasts in response to exercise, loading, and bone injury, we suggested that it may take part in mechanical stimuli-promoted proliferation and -inhibited differentiation in osteoblasts to facilitate bone formation.

Alternative splicing is a process that generates multiple mRNA variants from a single transcript, thereby enriching the mammalian proteome. This process is regulated by many factors such as cis-acting elements (RNA sequence elements) and trans-acting splicing factors (protein regulators) [35]. SR proteins are key regulators of alternative pre-mRNA splicing [36]. In most cases, SR proteins interact with exon splicing enhancer sequences to promote targeted exon inclusion. ASF/SF2 and SC35 are two of the best studied SR proteins, and they regulate pre-mRNA splicing on a genome-wide level. Our findings reported here showed that ASF/SF2 and SC35 were highly expressed in response to mechanical stimuli. This result indicated that mechanical stimuli may regulate alternative splicing by promoting the expression of ASF/SF2 and SC35. In regard of this, we hypothesized that ASF/SF2 and SC35 might be regulatory factors that influence MGF expression under mechanical stimuli. Therefore, we altered the expression of ASF/SF2 or SC35 and analysed its effect on the expression of MGF mRNA, and found that MGF expression was modulated by ASF/SF2. Given the above findings, we conclude that mechanical stimuli play a role as a specific activator of splicing to induce the expression of ASF/SF2, and then promote alternative *Igf1* gene splicing to generate the MGF splicing variant.

On the basis of our findings, the comprehensive role of MGF in bone metabolism, bone repair, and bone development should be investigated further. Due to its various effects on the physiological processes of osteoblasts, MGF shows great potential for the treatment of bone-associated diseases. In addition to mechanical stimuli, factors that influence the generation of MGF include hyperthermia, acidification, growth hormones, hypoxia, cell stress, and ischaemia, indicating the involvement of MGF in many physiological and pathological processes, its potential use as a diagnostic marker, and its possible therapeutic potential.

## Conclusions

**Figure 6.**
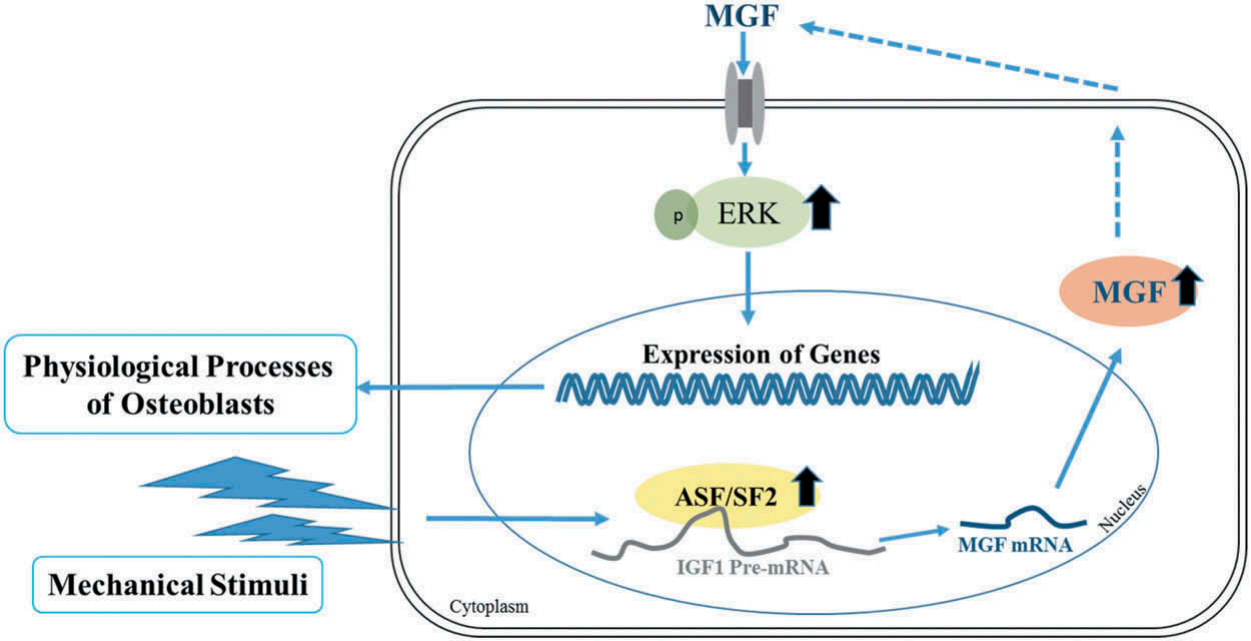
Cartoon depicting a splicing model under mechanical stimuli. ASF/SF2 proteins regulate the alternative splicing of the *Igf1* gene to generate MGF in osteoblasts in response to mechanical stimuli to regulate osteoblasts function.

Taken together, mechanical stimuli can influence a variety of physiological processes in osteoblasts, and induce alternative splicing of the *Igf1* gene to generate MGF. *In vitro*, MGF can regulate the proliferation, migration, and differentiation of osteoblasts. A splicing model under mechanical stimuli is shown in Figure 6, which infers the mechanism for the generation of mechanical stress-induced MGF expression. Considering that mechanical stimuli are involved in the induction of MGF production and the effects of MGF on the physiological responses of osteoblasts, MGF may have a critical function in bone growth and bone repair by stimulating cell proliferation, migration and promoting bone regeneration. Further studies are necessary to understand the mechanism by which MGF regulates bone growth and development, and to investigate the combined application of mechanical stimuli and MGF in bone repair.
